# Cognitive Task Domain Influences Cognitive-Motor Interference during Large-Magnitude Treadmill Stance Perturbations

**DOI:** 10.3390/s23187746

**Published:** 2023-09-08

**Authors:** Jessica Pitts, Lakshmi Kannan, Tanvi Bhatt

**Affiliations:** Department of Physical Therapy, University of Illinois at Chicago, 1919 W Taylor St., Chicago, IL 60612, USA

**Keywords:** reactive balance, dual-tasking, cognitive-motor interference, visuomotor

## Abstract

Reactive balance is postulated to be attentionally demanding, although it has been underexamined in dual-tasking (DT) conditions. Further, DT studies have mainly included only one cognitive task, leaving it unknown how different cognitive domains contribute to reactive balance. This study examined how DT affected reactive responses to large-magnitude perturbations and compared cognitive-motor interference (CMI) between cognitive tasks. A total of 20 young adults aged 18–35 (40% female; 25.6 ± 3.8 y) were exposed to treadmill support surface perturbations alone (single-task (ST)) and while completing four cognitive tasks: Target, Track, Auditory Clock Test (ACT), Letter Number Sequencing (LNS). Three perturbations were delivered over 30 s in each trial. Cognitive tasks were also performed while seated and standing (ST). Compared to ST, post-perturbation MOS was lower when performing Track, and cognitive performance was reduced on the Target task during DT (*p* < 0.05). There was a larger decline in overall (cognitive + motor) performance from ST for both of the visuomotor tasks compared to the ACT and LNS (*p* < 0.05). The highest CMI was observed for visuomotor tasks; real-life visuomotor tasks could increase fall risk during daily living, especially for individuals with difficulty attending to more than one task.

## 1. Introduction

A majority of real-life falls are unpredicted and commonly occur while performing more than one task (i.e., dual-tasking (DT)) [1]. When an unpredicted balance threat is encountered, the ability to recover and prevent falling depends on both physical ability and cognitive state [2]. DT paradigms provide insight into the cognitive demands of balance control by examining the ability to maintain posture while simultaneously performing a cognitive task [3]. Many studies have shown that during DT standing balance, there is an increase in postural sway or a decrease in cognitive performance (or both) compared to performance on each task individually [4,5,6]. This effect is known as cognitive-motor interference (CMI) [7] and suggests that balance control demands attention, such that the ability to maintain posture decreases when cognitive resources must be shared with another task [8,9]. However, most DT studies have focused on CMI during predicted conditions (i.e., volitional balance) and there is a limited understanding of CMI during unpredicted conditions (i.e., reactive balance).

Reactive balance control involves the rapid triggering of motor responses to re-establish stability of the center of mass (COM) relative to the base of support (BOS) following an unpredicted postural perturbation [10,11]. This is typically accomplished by the production of muscle torques at the hip or ankle that move the COM back to a position of stability (i.e., fixed-support strategies) or a change in the BOS dimensions by compensatory stepping/grasping (i.e., change-in-support strategies) [11,12]. Fixed-support responses are postulated to arise from spinal cord and brainstem neural loops and their fast response latency provides an important early defense against balance loss [13,14]. However, change-in-support strategies are necessary to prevent falling from large-magnitude perturbations (e.g., slips, trips) due to the stability gained by expanding the BOS [10,15]. Change-in-support strategies are postulated to have higher cortical center involvement than fixed-support strategies due to their complexity (i.e., shifting weight to one limb, maintaining single-stance balance, planning and initiating steps to the appropriate location) and likely involve a transcortical loop through the motor cortex, especially in the later phases of the response [13,16,17]. Therefore, the cognitive demands of reactive balance may be dependent on the magnitude of the perturbation and the strategy used to regain balance. 

DT studies have attempted to understand the cognitive demands of reactive balance by examining how a cognitive task (e.g., mental math, word list generation) affects the ability to recover from perturbations. When young adults were exposed to small magnitude perturbations that elicited fixed-support responses, DT did not affect the onset of initial postural responses [18,19], supporting the postulation that reactive balance responses originate reflexively from subcortical structures [20]. However, DT did affect the later phases of reactive balance, resulting in increased COM displacement and center of pressure (COP) excursions compared to single-task (ST) (i.e., responding only to the perturbation) [18,21]. These studies examining fixed-support strategies provide insight into the time course of cortical involvement in reactive balance and indicate that DT can impair the ability to maintain the COM state when facing instability. However, examining CMI during fixed-support responses does not explain how DT affects the ability to implement a stepping response to recover balance from large-magnitude perturbations during daily living. 

Compensatory stepping is often the preferred strategy to regain balance and occurs even at low perturbation magnitudes, especially in older adults [22,23,24], and when participants are instructed not to step [25]. Regardless, very few DT studies have examined compensatory stepping and have provided inconclusive evidence [23,26,27,28,29]. Some of these studies reported that DT impaired reactive balance (i.e., reduced stability, decreased step length, increased response time) in both younger and older adults [23,26,27], whereas others found little to no change in compensatory stepping characteristics [28,29]. There were also inconsistent findings within the cognitive performance domain, which either decreased or remained unchanged [23,26,27,28,29]. One reason for these inconsistent findings could be that studies included different types of cognitive tasks (e.g., mental math, word list generation, reaction time task), which can affect CMI patterns [30,31,32]. DT gait studies have demonstrated that mental math tasks affect gait speed, trunk control, and stride time variation more than other types of tasks (e.g., Stroop task, verbal fluency) [30,31,32]. However, to the authors’ knowledge, there have been no studies that have compared CMI between different cognitive tasks during reactive balance. It is suggested that there will be higher CMI between tasks that share more similar cognitive resources [8,33]. Therefore, comparing CMI between multiple cognitive tasks would provide insight into which cognitive domains play a larger role in reactive balance. Additionally, a DT paradigm with multiple cognitive tasks would help researchers and clinicians understand how fall risk is affected by different daily life cognitive tasks. 

The existing literature on DT reactive balance is also limited because it has primarily involved cognitive tasks in the working memory, verbal fluency, and executive function domains (e.g., mental math, word list generation, and trail making) [23,26]. These tasks are relevant to cognitive processes in real life, and it is certainly important to examine how they affect reactive balance. However, focusing on only the memory, language, and executive function domains may limit the generalizability of DT research to real-life scenarios because daily living tasks also involve other cognitive domains. Namely, the visuomotor domain is constantly engaged when navigating and interacting with the environment [34]. A few studies have examined how a visuomotor tracking task affected reactive responses to support surface translations, reporting increased COP excursions, increased step onset time, and more errors on the tracking task [18,35]. However, completing these tasks only required movements of the thumb, which involved minimal movement and no disruption of the visual field. A visuomotor task that better mimics real-life scenarios might be one that involves movements of the neck and head, similar to those made when visually scanning the environment or alerting attention to a postural threat. Such tasks have been implemented during DT walking in a few studies [36,37]. While moving the head to track a moving target or catch a target object, young adults showed increased gait variability and decreased cognitive performance (increased movement variance, decreased success rate) [36,37]. However, visuomotor tasks are yet to be examined during DT reactive balance. Incorporating visuomotor tasks with head movements would help explain the involvement of the visuomotor domain in reactive balance control and understand how head movements affect the ability to recover from perturbations.

The purpose of this study was to examine how DT affected the ability to respond to large-magnitude forward support surface translations (delivered via treadmill) that elicited a stepping response in healthy young adults. We hypothesized that DT would lead to CMI during perturbations, resulting in a lower post-perturbation margin of stability (MOS) and/or decrease in cognitive scores. Further, we aimed to compare CMI and task prioritization between different cognitive tasks (auditory clock test (ACT), letter-number sequencing (LNS), and two visuomotor tasks (Target, Track)). We expected that all types of cognitive tasks would result in CMI and have a negative effect on reactive stability and/or cognitive task performance during DT perturbations. However, this aim was exploratory in nature as the effect of different cognitive task domains on CMI during reactive balance has not previously been examined. Therefore, we did not formulate a hypothesis as to which type of cognitive task would result in the most CMI or affect prioritization pattern. This study will further the understanding of how real-life cognitive tasks affect fall risk in different scenarios and guide the future development of dual-task assessment or training protocols for reactive balance.

## 2. Materials and Methods

### 2.1. Participants

Young adults between the ages of 18–35 years were eligible to participate. This study was approved by the Institutional Review Board at the University of Illinois at Chicago (2022-0136) and participants were required to provide informed consent prior to participation. Participants were recruited by posting flyers at various college buildings and nearby bus stops and through word-of-mouth. After expressing interest in the study, potential participants were screened for study eligibility by a research member and were asked to provide their age and weight. Potential participants were also asked if they had any current medical conditions and if they had undergone a recent major surgery, bone fracture, or hospital admission. Participants were excluded if they self-reported any neurological, musculoskeletal, cardiopulmonary, or other systemic disorders that may have affected their ability to participate in the study. Additionally, participants were excluded if they self-reported any major surgery or bone fracture in the past six months, or had been admitted to the hospital in the past three months. Lastly, participants were excluded if their weight was >220 pounds, due to the weight threshold of the safety harness system. A total of 20 young adults met the eligibility criteria and participated in this study (40% female; 25.6 ± 3.8 y; 1.8 ± 0.1 m; 67.7 ± 13.6 kg; presented as mean ± SD). 

### 2.2. Experimental Protocol

#### 2.2.1. Treadmill Stance Perturbations

All study procedures were completed in a single testing session lasting about one hour. To minimize physical and mental fatigue, participants were offered rest breaks as needed throughout the study protocol. Participants were exposed to large forward support surface translations while standing on a motorized ActiveStep treadmill (Simbex, Lebanon, NH, USA). Each trial lasted 30 s, during which three perturbations were unexpectedly delivered at random intervals by sudden forward movement of the treadmill belt (displacement: 33 cm; velocity: 0.86 m/s; acceleration: 21.75 m/s^2^). This intensity of perturbation guaranteed that all participants required a stepping response to recover balance. This protocol was designed so that both reactive balance and cognitive performance could be evaluated over a longer-term period, rather than a singular event-based instance. Some previous papers examining DT reactive balance have evaluated short-duration trials (8 s) [23,27,28,29]; however, our paradigm might provide a more robust understanding of CMI during reactive balance and mimic real-life scenarios that involve long-duration cognitive tasks. These perturbations were delivered in ST (perturbation only, no cognitive task) and four DT conditions (perturbation while completing a concurrent cognitive task) in a randomized order with each condition completed once. A description of the cognitive tasks used during DT conditions can be found in the following section (“Cognitive Tasks”). Due to the rapid adaptation to perturbations observed after only a few exposures [38], participants were given a familiarization perturbation trial before testing to minimize the effect of adaptation on analyzed trials. For safety, participants wore a full-body safety harness connected to an overhead I-bar for the duration of the experiment to prevent them from contacting the ground should they experience a fall. 

#### 2.2.2. Cognitive Tasks

**Figure 1:**
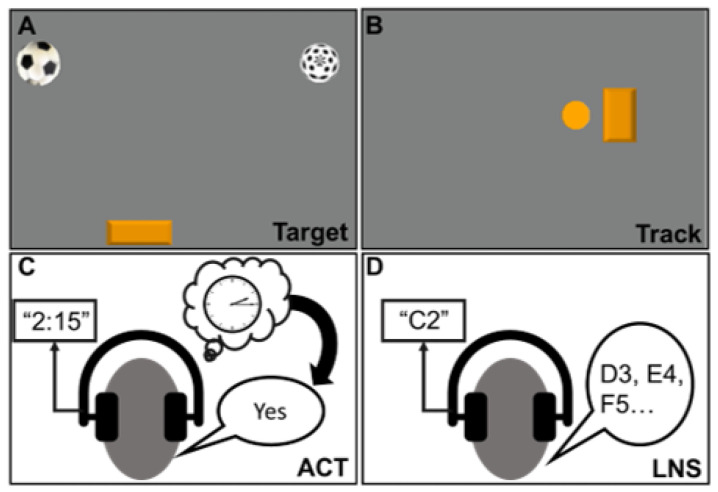
Cognitive tasks completed during large-magnitude support surface perturbations: Target (**A**), Track (**B**), auditory clock test (ACT) (**C**), and letter number sequencing (LNS) (**D**). For the two visuomotor tasks (Target, Track), participants wore an inertial-bead mouse on the top of their heads that controlled a cursor on the screen. During the Target task, participants controlled an orange paddle on the bottom of the screen and turned their heads left and right to catch a soccer ball that fell from the top of the screen, while avoiding the other “distractor” ball. During the Track task, the orange circle on the screen was computer-controlled and moved left to right in a sinusoid pattern. Participants controlled the orange rectangle on the screen and turned their heads left and right to track the movement of the circle. For the auditory clock test (ACT), participants heard a time of the day (e.g., “2:15”) and responded “yes” if the hour and minute hand both pointed to the same half (left or right) of the clockface or “no” if they pointed to opposite sides. For the letter number sequencing (LNS) task, participants heard a letter and number pair (e.g., C2) and responded by listing the next sequential letter and number (e.g., D3, E4, etc.).

Participants completed the following cognitive tasks in a randomized order during DT trials (Figure 1). Prior to DT trials, these cognitive tasks were also completed in ST (cognitive task only, no perturbation) while seated and standing on the same treadmill.

1. Target Game: The Target game was designed to engage the visuomotor cognitive domain. Participants looked at a screen directly in front of the treadmill and had an inertial-bead mouse (Therapy Mouse, Mobility Research, Phoenix, AZ, USA) secured on the top of their head that allowed them to control the mouse on the computer screen by rotating their head left and right (Figure 1A). For this task, participants controlled a paddle located on the bottom of the screen. The goal of the task was to move the paddle left and right to catch soccer balls that dropped from the top of the screen every two seconds. There was another ball with a different pattern that dropped from the top of the screen at the same rate, but participants were told to ignore this ball and catch only the soccer ball. Data regarding the coordinates of the soccer ball and the participant-controlled paddle were sampled at a rate of 100 Hz. Cognitive performance was determined by performance error, which was the average distance between the participant-controlled paddle and the soccer ball when it reached the bottom of the screen for all dropped balls. This task was custom-developed using Digital Rehab Research software, v. 1.88 [36]. Performance error was averaged over the entire 30 s trial duration. For all cognitive tasks, we chose to analyze cognitive performance over the entire trial duration because we considered the whole 30 s trial to be a DT scenario. Three perturbations were delivered during this period with each recovery period lasting ~5–7 s (compensatory stepping response(s)), allowing participants to re-establish a baseline state before the next perturbation was delivered. Thus, although perturbations were not continuously delivered, participants were engaged in the reactive balance task for a majority of the trial.
2. Track Game: The Track game was also designed to engage the visuomotor cognitive domain. Similar to the Target game, participants looked at a screen directly in front of the treadmill and wore an inertial-bead mouse on their heads. For this task, there was a computer-controlled ball that moved right and left in a sinusoid pattern (frequency = 0.4 Hz; amplitude = 80% of screen width) on the screen. Participants controlled a rectangle on the screen and were asked to rotate their heads right and left (using the inertial-bead mouse) to overlap the rectangle (controlled by head rotation) on top of the circle (computer-controlled motion) (Figure 1B). Cognitive performance was determined by the sum of errors, which was the total sum of overshoots and undershoots (i.e., difference in location between the computer-controlled target and participant-controlled rectangle) throughout the duration of the 30 s trial. This task was custom-developed using Digital Rehab Research software, v. 1.88 [36].
3. Auditory Clock Test (ACT): The ACT was designed to involve visuospatial working memory. For this task, participants wore a pair of headphones with a built-in microphone to record their verbal responses. Participants would hear a time of the day (e.g., 1:15, 2:45) and were asked to imagine a clock and verbally respond “yes” if the hour and minute hand of the clock were on the same half (right or left) of the clockface, or “no” if the hour and minute hand of the clock were on opposite halves of the clockface (Figure 1C). For example, if the participant heard “1:15”, both the hour and minute hands were facing the right half of the clockface, and the correct answer would be “yes”. Participants were given eight cues to respond to over a period of 30 s. Cognitive performance was determined by accuracy, which was calculated by dividing the number of correct responses by the total number of cues given (i.e., eight). This task was developed and delivered using DirectRT Research software, v. 2014.1.123 (Empirisoft, New York, NY, USA).
4. Letter Number Sequencing (LNS): The LNS was designed to engage the executive function domains, particularly, working memory and cognitive flexibility. For this task, participants also wore a pair of headphones, and verbal responses were recorded with a built-in microphone. Participants would hear a cue that consisted of a letter and number paired together (e.g., A5) and asked to continue the sequence by listing the next consecutive letter and number (i.e., B6, C7, D8, etc.) (Figure 1D). Participants were asked to continue listing the sequence until they heard another cue. Three cues were given over the 30 s period. Cognitive performance was determined by the number of correct responses. This task was developed and delivered using DirectRT Research software, v. 2014.1.123 (Empirisoft, New York, NY, USA).

### 2.3. Data Collection and Analysis

During perturbation trials, kinematic data were collected from an 8-camera motion capture system (Qualisys Motion Capture, Goteberg, Sweden) at a sampling frequency of 120 Hz. A total of 26 reflective markers were placed on the participant using a full-body Helen Hayes marker set. Individual marker trajectories were identified and gap-filled using Qualisys software, v. 2.9, and then full-body marker data were analyzed to calculate variables of interest using a custom MATLAB code (MathWorks, Natick, MA, USA). 

To quantify reactive balance performance, we calculated the margin of stability (MOS), which provides a measure of the position and velocity of the COM relative to the BOS at a specific time instance [39]. We calculated MOS at the instance of 0.3 s post-belt onset. This time was selected because we wanted to compare reactive stability between conditions when all participants had experienced a loss of balance and were in mid-swing of the single stance phase after initiating a recovery step, which we visually verified for each trial. This decision was based on previous evidence, which has indicated that the liftoff of the first compensatory step occurs around 0.21 s following perturbation onset during similar stance treadmill perturbations in young adults [40]. We confirmed this in the current sample and found that recovery step liftoff and touchdown occurred at 0.20 ± 0.04 s and 0.42 ± 0.07 s after perturbation onset, respectively. Additionally, calculating MOS at a fixed-time instance ensured that the movement of the treadmill belt (acceleration and position) was equivalent for all trials. Assessing MOS at an event-based instance such as recovery step liftoff or touchdown could affect the calculated MOS if the treadmill is in different phases of movement (e.g., accelerating/decelerating or moving constantly). We also calculated baseline MOS at the instant of perturbation onset (i.e., the time at which acceleration of the treadmill belt changed from 0 m/s^2^) to ensure that pre-perturbation stability was equivalent between all conditions. MOS was calculated using the following equation, in which COM_x_ and COM_v_ indicate the position and velocity of the COM in the anterior-posterior direction, “g” indicates the gravitational constant, “l” indicates the length of the leg, and BOS_x_ indicates the posterior border (i.e., heel) of the compensatory stepping foot:

(1) $$MOS={COM}_{x}+\frac{{COM}_{v}}{\sqrt{\frac{g}{l}}}-{\mathrm{BOS}}_{x}$$

MOS was further normalized by foot distance, which is the distance between standing foot toe and stepping foot heel at the given time instance, and thus ranges from −1 to 1. In this study, MOS values < 0 indicate that the COM is not stable relative to the BOS, and MOS values > 0 indicate that the COM is in a position of stability. For data analysis, MOS was averaged between the three perturbations within each trial. All time events were identified visually using marker trajectory data in Qualisys software, v. 2.9.

To test the difference in reactive balance performance between ST and DT, we used a repeated measures ANOVA to examine the overall effect of task (ST, ACT, LNS, Target, Track) on MOS at baseline and post-perturbation. To ensure that there was no effect of adaptation on MOS, we also conducted an ANOVA to examine the effect of trial order on MOS. To test differences in cognitive performance between ST and DT, we used a repeated measures ANOVA to compare ST seated, ST standing, and DT. Further, we calculated the percent change (%Δ) in MOS and cognitive performance between ST and each DT condition using (single-dual)/single × 100. We also examined the total %Δ in performance by taking the sum of the %Δ in MOS and cognitive performance for each task. We then compared the %Δ in performance (cognitive, motor, and combined) between all tasks (Target, Track, ACT, LNS) using a repeated measures ANOVA. If any significant main effects were observed, we conducted follow-up post hoc tests with a Bonferroni correction. All statistical analyses were performed using SPSS v. 25 (IBM Corporation, New York, NY, USA) with an alpha level set at 0.05.

### 2.4. Power Analysis

We used previously published data from our laboratory to conduct a power analysis for the present study [26]. This study compared reactive stability (quantified by the COM position) between ST and DT treadmill stance perturbations with a working memory task. Based on mean differences in reactive stability, our sample size of n = 20 could detect differences between ST and DT with >98% power, based on a significance level of 0.05. The power analysis was conducted using G*Power v. 3.1. 

## 3. Results

**Figure 2:**
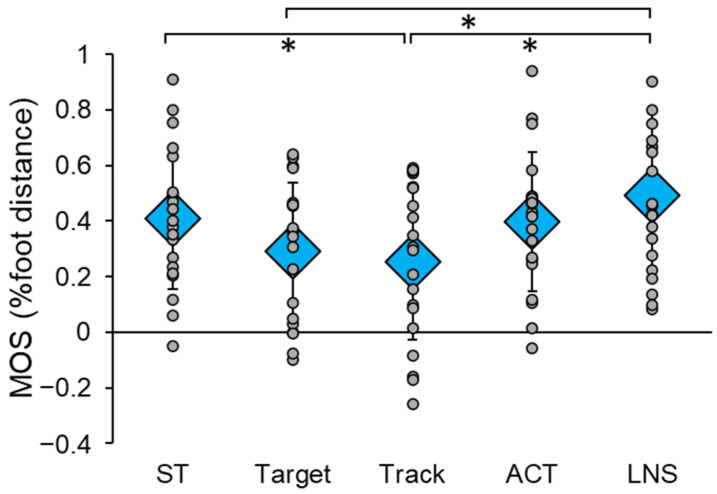
Reactive balance performance in single-task (ST) and dual-task (DT) conditions, measured by post-perturbation margin of stability (MOS). The ST condition consisted of three perturbations given at random times within 30 s. The DT conditions involved the concurrent performance of four different cognitive tasks during these perturbations: Target, Track, auditory clock test (ACT), and letter number sequencing (LNS). Circular markers indicate the MOS of each individual participant; diamond markers indicate the mean MOS for all participants in each condition. * indicates *p* < 0.05.

DT had a significant main effect on post-perturbation MOS (F(4,76) = 7.011, *p* < 0.001, η_p_^2^ = 0.270) (Figure 2). Specifically, post hoc tests revealed that MOS was significantly lower in Track compared to ST (*p* = 0.049) and LNS (*p* = 0.001), and significantly lower in Target compared to LNS (*p* = 0.027). There was no significant effect of trial order on post-perturbation MOS (F(5,94) = 0.647, *p* = 0.665, η_p_^2^ = 0.033). Additionally, there were no significant differences in baseline MOS between conditions (F(4,76) = 0.503, *p* = 0.733, η_p_^2^ = 0.026), indicating that pre-perturbation stability while standing was not affected by the performance of any cognitive task.

**Figure 3:**
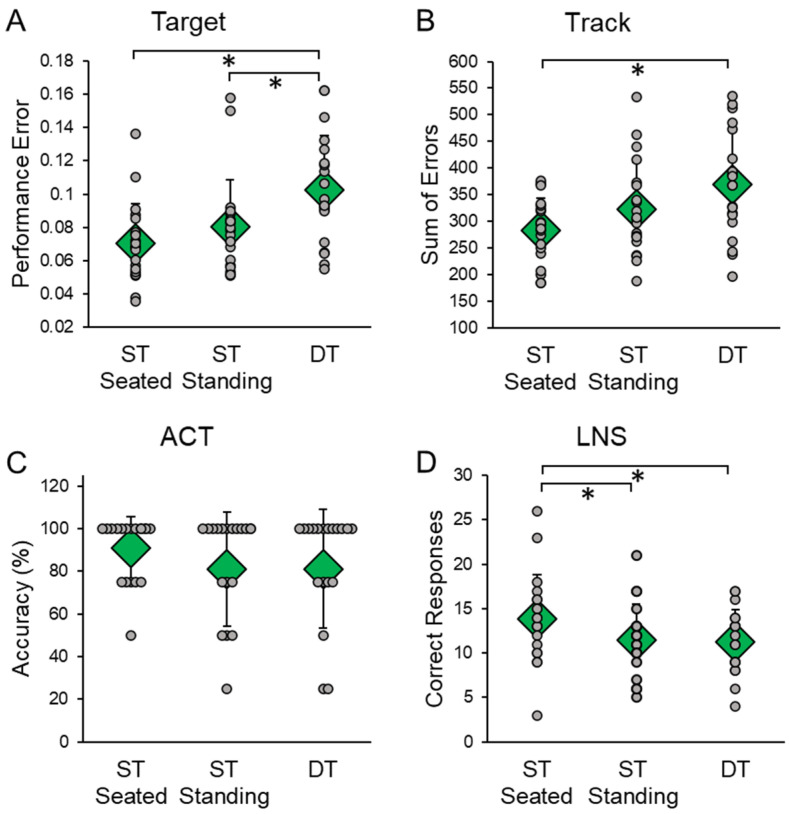
Cognitive scores in single-task (ST) and dual-task (DT) conditions. Cognitive tasks included the (**A**) Target, (**B**) Track, (**C**) auditory clock test (ACT), and (**D**) letter number sequencing (LNS) tasks. ST conditions were completed while seated and standing. Cognitive tasks were completed for 30 s; in ST only the cognitive task was completed, in DT three unexpected treadmill support surface perturbations were delivered. Circular markers indicate the performance of each individual participant; diamond markers indicate the mean performance for all participants in each condition. * indicates *p* < 0.05.

DT significantly affected cognitive performance on the Target (F(2,38) = 7.206, *p* = 0.002, η_p_^2^ = 0.275) (Figure 3A). Pairwise comparisons indicated that performance error was significantly higher during DT than during ST seated (*p* = 0.009) and ST standing (*p* = 0.040). DT also affected cognitive performance on the Track (F(2,38) = 6.369, *p* = 0.004, η_p_^2^ = 0.251) (Figure 3B). Pairwise comparisons indicated that the sum of errors was significantly higher during DT than ST seated (*p* = 0.010). DT also affected cognitive performance on the LNS task (F(2,38) = 8.265, *p* = 0.001, η_p_^2^ = 0.303) (Figure 3D). Pairwise comparisons indicated that the number of correct responses was significantly higher during ST seated than ST standing (*p* = 0.008) and DT (*p* = 0.008). DT did not affect cognitive performance on the ACT (F(2,38) = 0.603, *p* = 0.552, η_p_^2^ = 0.031) (Figure 3C). 

**Figure 4:**
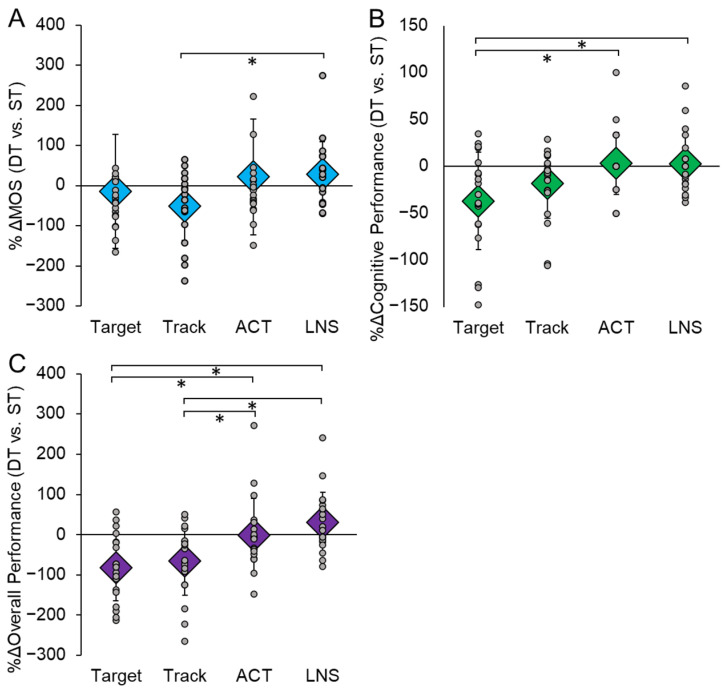
The percent change (%Δ) in motor (**A**) and cognitive (**B**) performance in each dual-task (DT) condition compared to single-task (ST) performance. Motor performance was quantified by the post-perturbation margin of stability (MOS) and cognitive performance was determined by the cognitive outcome for each specific task. We also took the sum of the %Δ in motor and cognitive performance for each task to investigate the overall decline in performance (**C**). A larger negative value indicates a greater reduction in performance during DT compared to ST. Circular markers indicate the %Δ in performance of each individual participant; diamond markers indicate the mean %Δ in performance for all participants in each condition. * indicates *p* < 0.05.

Additionally, we examined the percent change (%Δ) in both motor and cognitive performance during each DT condition compared to ST. For motor performance, there was a significant main effect of the type of task on the %Δ in MOS (F(3,54) = 4.635, *p* = 0.006, η_p_^2^ = 0.205) (Figure 4A). Compared to ST performance, there was a greater reduction in MOS while performing Target compared to LNS (*p* = 0.010). There was also a main effect of the type of task on the %Δ in cognitive performance (F(3,57) = 5.037, *p* = 0.004, η_p_^2^ = 0.210) (Figure 4B). Compared to ST performance, there was a larger reduction in cognitive performance during DT on the Target compared to the ACT (*p* = 0.017) and LNS (*p* = 0.022). Lastly, there was a main effect of the type of task on %Δ in overall performance (cognitive + motor) (F(3,57) = 10.547, *p* < 0.001, η_p_^2^ = 0.369). There was a greater reduction in overall performance during both the Target and Track tasks compared to the ACT and LNS tasks (*p* < 0.05) (Figure 4C).

## 4. Discussion

The purpose of this study was to examine how DT affected the ability to respond to large-magnitude perturbations requiring a reactive stepping response in young adults. Further, we examined if the type of cognitive task affected CMI during DT reactive balance. We found that the type of cognitive task was significant in determining the magnitude of CMI. Visuomotor tasks resulted in the highest CMI and had a significant negative effect on both the ability to implement a reactive stepping response (lower post-perturbation MOS) and cognitive performance (increased movement error), resulting in a mutual interference pattern. The ACT and LNS did not have a significant negative effect on reactive balance outcomes or cognitive performance. These results indicate that reactive stepping responses may primarily rely on cognitive resources in the visuomotor domain. 

We found that reactive stepping was impaired when performing the visuomotor tasks, compared to ST. Post-perturbation, the COM was closer to the backward limits of stability imposed by the BOS, suggesting that the COM was in a less stable position. According to DT theories (e.g., capacity sharing theory), this CMI occurs because the cognitive and motor tasks share overlapping neural resources, which are limited in supply, leading to a deterioration in performance on either or both tasks [8,33]. In support of this theory, neuroimaging studies have shown that similar brain areas (supplementary motor area, parietal lobes, and cerebellar regions) are activated both when imagining a slip perturbation [41] and performing a visuomotor task (i.e., drawing a shape within constraining borders) [42]. Therefore, our results provide preliminary evidence that the visuomotor domain contributes to reactive balance and may play a large role in rapidly initiating and implementing a stability-restoring response, more so than cognitive processes involved in the LNS (working memory, cognitive flexibility) or ACT (visuospatial memory). Although DT studies have not widely examined the relationship between reactive balance and the visuomotor domain, some studies have used tasks similar to the Target and Track during unperturbed gait to examine the role of the visuomotor domain in controlling posture [36,37,43]. These studies similarly found that DT resulted in mutual interference; there was a deterioration in motor performance (increased variability of step length, stride time [36,43], and center of pressure displacement [37]), and cognitive performance (Target: delayed initiation of head rotations, increased movement duration, decreased success rate [37,43]; Track: increased amplitude variability, increased total error [36]) during DT gait compared to ST.

The visuomotor domain may be particularly important during postural tasks because visual processing of the environment allows the CNS to monitor the relative position of the body and modify movements based on environmental conditions (e.g., barriers, obstacles, or uneven surfaces) [44]. Visual processing could be important to modulate posture both in anticipation of a balance threat (i.e., modifying gait when walking on a slippery surface) and when a balance disturbance occurs. Post-perturbation, visual information is integrated with proprioceptive and vestibular information to help detect and characterize the perturbation and implement a corrective stepping response that is appropriate in the given environment (e.g., step location and trajectory) [44]. The visuomotor tasks may have caused interference with reactive balance because they required continual movements of the head to track or catch the moving target [43], leaving few cognitive resources available for visual processing of the postural state. However, it is likely that the reduction in reactive stability during visuomotor tasks was not only due to the head movements required by the tasks. We found that there was no difference in baseline stability while performing the visuomotor tasks, which suggests that the effects of head movements alone on stability are minimal, and the reduction in post-perturbation stability stemmed from the engagement of the visuomotor cognitive domain. However, future studies involving more types of visuomotor and visuospatial tasks (which do not require head movement) would be necessary to confirm this postulation.

Additionally, cognitive resources in the visuomotor domain may be involved in accessing or updating internal models stored in the CNS for reactive balance. As perturbations are repeated, there are trial-by-trial increases in stability [38] and improvements in associated variables (e.g., compensatory step length, number of compensatory steps) [45], which are postulated to occur as sensorimotor feedback is used to update an internal model for movement planning [46,47]. In subsequent perturbations, this internal model is accessed to plan appropriate corrections to reactive balance based on movement errors from the preceding trials [48,49]. It is possible that the updating of, or access to, this internal model relies on cognitive resources, particularly in the visuomotor domain. With fewer cognitive resources available while performing visuomotor tasks, individuals may not have been able to effectively access their internal model, which interfered with the ability to integrate sensorimotor feedback and make trial-by-trial modifications based on performance in the previous trial.

The LNS resulted in minimal CMI and did not have a large effect on reactive balance performance or cognitive performance. LNS performance did decline during DT trials (i.e., motor-related cognitive interference) compared to ST seated performance. However, LNS performance was not different between standing trials and perturbation trials. Thus, the resources involved in performing the LNS task (working memory and/or cognitive flexibility) may not be specific to reactive balance but rather be involved generally for both volitional (i.e., standing balance) and reactive balance control. Previous neuroimaging studies have shown the activation of the supplementary motor area and other frontoparietal regions [50,51] during both executive function/working memory tasks and slip imagery [52]. Additionally, previous DT studies have found that working memory tasks result in increased postural sway during quiet standing [4,5]. Thus, the LNS-related domains could contribute to both volitional and reactive balance control and be involved in the planning or modulating of motor responses based on sensorimotor integration, environmental layout, and prior exposure. However, our results suggest that when cognitive resources need to be shared between a balance task and the LNS, the CNS may prioritize these resources toward motor performance, leaving limited resources for cognitive performance. This prioritization could have been influenced by a number of factors, including the perceived difficulty/complexity of each task, motivation, and attentional demands [53,54]. 

Interestingly, this trend of lower performance in standing vs. sitting was also observed for the visuomotor tasks (Track, Target). This may have occurred because the stability requirements of an upright stance are greater than those of sitting [55,56] and thus may be more demanding of cognitive resources. A previous study found similar results, in which the cognitive performance of a working memory task was higher while sitting than standing [5]. Therefore, our results suggest that even quiet standing could be cognitively demanding enough to result in CMI and a reduction in cognitive performance. 

There was no difference in performance on the ACT between ST and DT, indicating that the ACT may not have been attentionally demanding enough to cause interference between the cognitive and motor tasks. The ACT involved visuospatial memory, which is also involved in the Target and Track tasks. However, we may not have observed the same CMI effect on the ACT because, unlike the Target and Track tasks, the ACT did not require any real-time visual processing of the environment. Further, the Target and Track tasks may have required more cognitive resources because they also required the processing of timely visual feedback to make appropriate corrective movements to the head. 

Our findings are significant because they suggest that fall risk during real-life DT scenarios depends on the type of cognitive task being performed. While reactive balance may be prioritized (or not affected) during certain tasks, other domains of cognitive tasks that cause more CMI could impair the ability to regain stability and lead to increased fall risk, especially for older adults or individuals with other pathological conditions that impair balance who have lower stability [57] and higher CMI [32,58,59] than young adults. Visuomotor tasks are involved continuously during daily living (e.g., turning the head to watch traffic while crossing the road, especially in the presence of environmental hazards such as icy streets or construction), and could impose a higher fall risk than other types of tasks should a loss of balance occur. Clinicians and researchers could be aware of the heightened risk in such situations and investigate potential preventative measures to reduce CMI during reactive balance and other postural tasks. One potential mechanism to reduce CMI and improve postural control is incorporating DT conditions into conventional training paradigms, which can increase their effectiveness in healthy and neurologically impaired populations [60,61]. However, DT training paradigms have largely involved only volitional balance tasks [62,63,64], which do not prepare individuals to respond to unexpected balance threats. Future researchers may want to investigate the effectiveness of perturbation-based reactive balance training in combination with DT conditions for reducing fall risk, especially including challenging conditions such as visuomotor tasks. Additionally, it is possible that assessing CMI while performing visuomotor tasks during DT reactive balance could improve the accuracy of fall risk assessment in older adults by assessing individuals in a challenging CMI condition. On the other hand, DT paradigms for fall risk assessment and training may not benefit from including tasks such as the ACT, which we did not find to cause interference with reactive balance.

The visuomotor tasks employed in the current study involved a human–computer interaction, which was initiated via head movements. However, future researchers could consider implementing other systems that might provide a more in-depth examination of the contribution of the visuomotor domain during DT reactive balance. For instance, several systems and programming technologies allow for the capture and complex analysis of eye-tracking metrics via human–computer interactions [65,66]. Examining eye movements during different visuomotor tasks could potentially be more sensitive than head movements for calculating CMI. Eye tracking metrics can indicate arousal, decision-making effort, and attention switching [67] and are evidenced to be one of the most sensitive physiological indicators of cognitive load [68]. Complex eye-tracking analyses could be used to develop new algorithms using programming technologies such as LINQ, thus enabling the calculation of cognitive load through the development of new source codes or the ability to perform readability testing of algorithm description tools [69]. However, eye-tracking systems alone are not able to directly record neural activity during postural tasks; thus, systems such as non-invasive electroencephalography (EEG), which allows for the mobile recording of cortical activity, could also be employed to provide insight into the time course of cortical activity/cognitive load during perturbation exposure [70]. Complex EEG analyses could also be used to perform source localization and examine which neural regions are active during ST and DT reactive balance [71]. 

DT assessments and training could also benefit from the implementation of virtual reality/augmented reality (VR/AR) or cost-effective brain–computer interfaces. VR/AR systems provide an immersive environment that simulates what might be experienced in real life, such as walking down a busy street and avoiding different postural threats [72,73]. As a result, incorporating VR/AR settings into balance assessment or training can mimic realistic DT scenarios while also being engaging, thus increasing intrinsic participant motivation and rehabilitation adherence [74,75]. Additionally, VR/AR balance assessments could be used to conduct vision screenings, through the ability to measure visual acuity and identify abnormal eye movements in a dynamic environment [76,77]. Rehabilitation engagement could also be increased via brain–computer interfaces, which record real-time neural signals (e.g., via EEG) and transform motor intent into output commands [78]. These brain–computer interfaces allow for the development of interactive videogames/exergames, and are becoming increasingly popular in rehabilitation for populations such as those with stroke or spinal cord injury, with higher effectiveness than conventional therapies for restoring motor function [79,80]. Such advances in systems such as VR/AR and brain–computer interfaces may also benefit rehabilitation by increasing participant self-confidence and self-efficacy [81,82]. Previous studies have shown that incorporating “gamified” systems and computer/programming training into learning modules can improve self-efficacy more than standard instruction alone [83,84]; thus, it is likely that such systems could have a similar effect on self-efficacy during cognitive-motor rehabilitation.

Thus, in addition to implications for the clinical field, our results could also be useful for researchers in the engineering sciences. Particularly, our study underscores the importance of considering the type of cognitive task during DT reactive balance paradigms, with visuomotor tasks inducing the highest CMI. This opens the door to utilizing more advanced technologies and analytical methods for examining cognitive performance and cognitive load during DT, especially given more recent advances in eye tracking and VR/AR systems. These technologies might provide a more sensitive measurement of visuomotor task performance and mimic realistic DT scenarios, yet would require more advanced programming knowledge to set up and analyze. Additionally, researchers with knowledge of signal processing could analyze neural activity via mobile neuroimaging during DT reactive balance, which could provide a more detailed indicator of the magnitude and time course of cognitive load during different types of cognitive tasks. Lastly, engineers may want to apply these results toward developing novel dual-task assessments and training paradigms that involve exergaming interfaces. Such tasks could engage multiple cognitive domains and be interactive and engaging for enhanced training satisfaction and compliance. 

This study had some limitations which should be considered. First, we exposed participants to many perturbations so that we could compare reactive balance responses between multiple DT conditions. It is possible that this resulted in adaptation to the perturbation and affected how attention was allocated between reactive balance and the cognitive task. However, we found that there was no effect of trial order on the MOS, which indicates that adaptation likely did not affect study findings. Additionally, it is possible that due to the repeated design of the study, participants experienced some mental or physical fatigue that affected their task performance. However, due to the non-significant trial order effect, short duration (<1 h) of the study protocol, and this being a sample of healthy young adults, we expect that these effects were minimal. Future studies could consider using a counter-balanced design to compare CMI between different cognitive tasks on novel perturbation exposure, which would limit adaptation and fatigue. Additionally, even though perturbations were temporally unpredictable, they could still be anticipated. It is possible that attention may be allocated differently in real-life scenarios when perturbations are novel and unexpected. However, in a preliminary analysis of the present study, we found that DT had a similar effect on MOS whether calculating the average MOS between three perturbations or when only examining the first perturbation in each trial. Thus, our findings could still be generalizable to novel perturbations. Another limitation is that we only exposed participants to one intensity of stance perturbations. Future studies may include more perturbation intensities to reduce anticipation and examine how DT affects the ability to scale responses to the intensity of the perturbation. Further, it could be seen as a limitation that we calculated cognitive performance across the entire 30 s trial, even though perturbations were not delivered continuously for this entire time. We calculated cognitive performance in this manner because this was a preliminary investigation into CMI during reactive balance and it is unknown how long CMI effects last following a perturbation. Further, participants were engaged in the reactive balance task for a majority of the 30 s trial. Future investigations could evaluate the time course of CMI effects following (and anticipating) perturbations. Lastly, we only included young adults in this study. Future studies should examine how CMI is affected by the type of cognitive task in different populations.

## 5. Conclusions

In conclusion, we found that the visuomotor, working memory, and cognitive flexibility domains could all be involved in reactive balance. The highest CMI resulted from visuomotor tasks, which interfered with the ability to recover stability following large-magnitude perturbations and could increase fall risk during real-life losses of balance. Future researchers should consider the influence of the cognitive task domain on reactive balance in older adults and pathological populations at increased risk of falls.

## Data Availability

The data presented in this study are available on request from the corresponding author.
